# Case of Lobulated Polypus of the Os Uteri

**Published:** 1833-05

**Authors:** J. F. Palmer

**Affiliations:** Surgeon to the Westminster General Dispensary. 38, Golden square.


### CASE OF LOBULATED POLYPUS OF THE OS UTERI.

*To the Editors of the London Medical and Physical Journal.*

Dear Sirs: As the following is not a very common case of polypus, it may be worthy of being shortly recorded.

Mrs. ——, æt. thirty-eight, was married at seventeen, and has had six children, the youngest of whom is now seven years of age. Two years ago she had a miscarriage, attended with rather a considerable degree of pain, hemorrhage, and followed by an abundant discharge for the succeeding six months; which about this time was much increased by the addition of a gonorrhœa; and her husband, who was a profligate fellow, inflicted, in addition, some manual injury upon the parts. The ardor urinæ, the discharge, the occasional losses of blood, and the excoriation of the adjacent parts, which were thus induced, continued for a period of eighteen months, and materially impaired her health.

I was requested, by Dr. Davies, on the 7th of November last, to ascertain, by examination, the cause of these symptoms, which had been found intractable under the ordinary methods of treatment. The polypus which I found consisted of several lobes, projecting about an inch into the vagina, and was attached to the posterior half of the circumference of the os uteri; the anterior half, and the cervix, being equally free from any unusual degree of induration or tenderness, and presenting none of the characters of a malignant disease. The form, however, and general characters of the tumor itself were considered by Drs. Davies and Lee to be of a very suspicious nature, especially as there was an evident ulceration on its anterior part, which occasioned the examination to be attended with more pain and hemorrhage than might have been anticipated from the examination of a common polypus.

The polypus being broadly based, and without any evident cervix, I was doubtful whether it would be practicable to employ the ligature; but, with the double canula, I experienced less difficulty than I expected. It was applied on the 12th of November, and came away on the 22d of the same month, without the production of a single unfavorable symptom. In about three weeks, the sore had healed, the discharge ceased, and the health of my patient was re-established. There remained, however, a permanent deficiency of substance on that side of the circumference of the os uteri, and, for some weeks afterwards, a considerable tenderness of the cervix.

I am not disposed to regard this case as affording any new light upon the subject of polypus; it only affords another proof of what is already generally known, namely, the propriety of making an early examination, the expediency of operating, and the facility and little inconvenience attending the operation; and, at the same time, that it may serve the purpose of future inquirers into the varieties of this disease.

J. F. Palmer,
Surgeon to the Westminster General Dispensary.

38, *Golden square.*

